# LIV-1 Promotes Prostate Cancer Epithelial-to-Mesenchymal Transition and Metastasis through HB-EGF Shedding and EGFR-Mediated ERK Signaling

**DOI:** 10.1371/journal.pone.0027720

**Published:** 2011-11-16

**Authors:** Hui-Wen Lue, Xiaojian Yang, Ruoxiang Wang, Weiping Qian, Roy Z. H. Xu, Robert Lyles, Adeboye O. Osunkoya, Binhua P. Zhou, Robert L. Vessella, Majd Zayzafoon, Zhi-Ren Liu, Haiyen E. Zhau, Leland W. K. Chung

**Affiliations:** 1 Department of Biology, Georgia State University, Atlanta, Georgia, United States of America; 2 Department of Medicine, Samuel Oschin Comprehensive Cancer Institute, Cedars-Sinai Medical Center, Los Angeles, California, United States of America; 3 Department of Urology, Emory University School of Medicine, Atlanta, Georgia, United States of America; 4 Department of Biostatistics, Emory University School of Public Health, Atlanta, Georgia, United States of America; 5 Department of Pathology, Emory University School of Medicine, Atlanta, Georgia, United States of America; 6 The Sealy Center for Cancer Cell Biology, University of Texas Medical Branch, Galveston, Texas, United States of America; 7 Department of Urology, University of Washington, Seattle, Washington, United States of America; 8 Department of Pathology, University of Alabama at Birmingham, Birmingham, Alabama, United States of America; 9 Department of Urology, Xijing Hospital, The Fourth Military Medical University, Xi'an, China; Penn State Hershey Cancer Institute, United States of America

## Abstract

LIV-1, a zinc transporter, is an effector molecule downstream from soluble growth factors. This protein has been shown to promote epithelial-to-mesenchymal transition (EMT) in human pancreatic, breast, and prostate cancer cells. Despite the implication of LIV-1 in cancer growth and metastasis, there has been no study to determine the role of LIV-1 in prostate cancer progression. Moreover, there was no clear delineation of the molecular mechanism underlying LIV-1 function in cancer cells. In the present communication, we found increased LIV-1 expression in benign, PIN, primary and bone metastatic human prostate cancer. We characterized the mechanism by which LIV-1 drives human prostate cancer EMT in an androgen-refractory prostate cancer cells (ARCaP) prostate cancer bone metastasis model. LIV-1, when overexpressed in ARCaP_E_ (derivative cells of ARCaP with epithelial phenotype) cells, promoted EMT irreversibly. LIV-1 overexpressed ARCaP_E_ cells had elevated levels of HB-EGF and matrix metalloproteinase (MMP) 2 and MMP 9 proteolytic enzyme activities, without affecting intracellular zinc concentration. The activation of MMPs resulted in the shedding of heparin binding-epidermal growth factor (HB-EGF) from ARCaP_E_ cells that elicited constitutive epidermal growth factor receptor (EGFR) phosphorylation and its downstream extracellular signal regulated kinase (ERK) signaling. These results suggest that LIV-1 is involved in prostate cancer progression as an intracellular target of growth factor receptor signaling which promoted EMT and cancer metastasis. LIV-1 could be an attractive therapeutic target for the eradication of pre-existing human prostate cancer and bone and soft tissue metastases.

## Introduction

LIV-1, a cell surface protein and a candidate mediator of the growth factor-elicited signaling molecule, has been associated with several important biologic processes by serving as a transporter for zinc and other ions [1], [2], [3], [4], [5]. As a prototype of the LIV-1 subfamily of ZIP metal transporters [5], [6], LIV-1 shares secondary structure with ZIP transporters and may have the ability to transport metal ions. LIV-1 was shown to be a mediator downstream from signal transducer and activator of transcription 3 (STAT3) and Snail, cooperating with Snail in the repression of epithelial marker E-cadherin (E-cad) gene transcription [7]. LIV-1 was also shown to be an interacting partner for the estrogen receptor (ER) in hormone-sensitive tissues [3], [8]. In the ER-positive ZR-75-1 breast cancer cell line, LIV-1 transcription is induced by estrogens [9]. In breast tumors, LIV-1 expression is associated with ER status [10], [11], and is positively correlated with the spread of cancer to regional lymph nodes [12]. In cervical cancer, expression of LIV-1 was shown to be higher in tumor than normal tissues; RNAi-mediated suppression of LIV-1 significantly inhibited cell proliferation, colony formation, and reduced the migratory and invasive ability of the HeLa cells [13]. LIV-1 has also been reported to be elevated in clinical pancreatic carcinoma and induced EMT in pancreatic cancer cells [14]. In zebrafish, LIV-1 is essential for the nuclear localization of Snail, a master transcription factor promoting epithelial to mesenchymal transition (EMT), causing migration of gastrula organizing cells [15]. LIV-1 thus is an obligatory co-factor regulating EMT-associated genes [14], [15], [16].

The potential diagnostic and prognostic value of LIV-1 in human prostate cancer has not been investigated. Since zinc plays important roles in the maintenance of prostate epithelial cell homeostasis [17], and Snail is a key transcription factor controlling prostate cancer cell EMT [18], [19], [20], LIV-1 may be an active participant in the promotion of EMT during prostate cancer progression and bone metastasis. In this study, we determined the level of LIV-1 in human prostate cancer cell lines and clinical tissue specimens to define the relationship between LIV-1 and prostate cancer progression and metastasis. The ARCaP human prostate cancer progression cell model was used to evaluate the role of LIV-1. Our study found that LIV-1 overexpression promotes prostate cancer cell EMT and facilitates its metastasis to bone and soft tissues. Further mechanistic investigation revealed that LIV-1 overexpression could upregulate HB-EGF and MMP2 and MMP9 expression. The latter could enzymatically cleave membrane-bound HB-EGF, to produce soluble HB-EGF that constitutively activated EGFR via increased EGFR phosphorylation and its downstream ERK signaling. The results from this study demonstrate that abnormally enhanced LIV-1 expression is a marker of prostate cancer progression, and activated LIV-1 is responsible for constitutive activation of EGFR which drives EMT. LIV-1 could be an attractive new therapeutic target for the inhibition of prostate cancer EMT and bone and soft tissue metastases.

## Materials and Methods

### Ethics statement

All animal work was conducted according to relevant national and international guidelines, and was approved by the Institutional Animal Care and Use Committee (IACUC) of Emory University School of Medicine (Permit number 254-2008).

### Cell lines and cell culture

Human prostate cancer ARCaP_E_ and ARCaP_M_ cells (derivative cells of ARCaP with epithelial and mesenchymal phenotype, respectively) were established in our laboratory [21]. The cells were cultured in T-medium (Invitrogen, Carlsbad, CA) supplemented with 5% fetal bovine serum (FBS, Atlanta Biologicals, Lawrenceville, GA). Human embryonic kidney HEK293 cells were obtained from American Type Culture Collection (Manassas, VA) and cultured in DMEM (Invitrogen) supplemented with 10% FBS. RPMI-1640 was purchased from Invitrogen (Carlsbad, CA). All the culture media were supplemented with penicillin (100 U/ml) and streptomycin (100 µg/ml). Cell cultures were maintained at 37°C in a humidified atmosphere supplemented with 5% CO_2_.

### Antibodies and reagents

Polyclonal rabbit antibody against LIV-1 was generated in our laboratory. Rabbits were immunized by standard immunization protocol with conjugated peptide KLH-CPDHDSDSSGKDPRNS, corresponding to residues 146-161 of the LIV-1 protein (GenBank accession number NM_012319). Blood was taken 2 weeks after the fourth boost and IgG were purified and tested for specific immune reactivity. Polyclonal antibody to E-cadherin (E-cad), monoclonal antibody to vimentin and phospho-EGFR antibody were purchased from Santa Cruz Biotechnology (Santa Cruz, CA). Monoclonal antibody to N-cadherin (N-cad) and EGFR were from BD Transduction Laboratories (San Diego, CA). Antibodies to p44/42 MAP kinase and the phosphorylated isoforms were from Cell Signaling Technology (Danvers, MA). Monoclonal antibody to β-actin was from Sigma-Aldrich (St. Louis, MO).

Insulin-like growth factor-1 (IGF-1) and transforming growth factor-β1 (TGF-β1) were purchased from Diagnostic Systems Laboratories (Webster, TX). Epidermal growth factor (EGF) was from R&D Systems (Minneapolis, MN). Tyrphostin AG1478, U0126 and MMP2/9 inhibitor III were obtained from Alomone labs (Jerusalem, Israel), Cell Signaling Technology (Danvers, MA), and Calbiochem (Darmstadt, Germany), respectively.

### Transfection

Full-length coding region for human LIV-1 cDNA was cloned and confirmed by DNA sequencing. The cDNA was then cloned downstream from a cytomegalovirus early promoter in the mammalian expression vector pcDNA3.1/V5-His (Invitrogen). HEK293 and ARCaP_E_ cells were seeded at 3×10^5^ cells per well in 6-well plates 24 hours before transfection. The cells were transfected with 4 µg of the LIV-1 expression construct using 8 µl Lipofectamine 2000 (Invitrogen). To isolate clones stably overexpressing LIV-1, transfected ARCaP_E_ cells were treated with G418 (600 µg/ml) 2 days after the transfection. Four individual clones overexpressing LIV-1 protein (LIV#8, #12, #14 and #17) and two clones transfected with control vector (con1 and con2) were used for the studies.

### siRNA knockdown

LIV-1 siRNA was purchased from Invitrogen. ARCaP_M_ cells were seeded at 3×10^5^ cells per well in 6-well plates for 24 hrs. The cells were transfected with 2.5 µl of 20 µM LIV-1 siRNA or equal amount of universal control siRNA, using 8 µl Lipofectamine 2000 per well. Cells were harvested and assayed 48 hours after transfection.

### Semiquantitative expression analysis with reverse transcription-polymerase chain reaction (RT-PCR)

Total RNA was isolated from cultured cells using the RNeasy Mini kit (Qiagen, Valencia, CA). From each sample, equal amount of RNA (2 µg) was used in first–strand cDNA synthesis reaction with the Superscript First-Strand cDNA Synthesis kit (Invitrogen). Equal volumes of cDNA (3 µl) from each reaction were used for PCR analysis using gene-specific oligonucleotide primer pairs: 5′-GCAATGGCGAGGAAGTTATCT-3′ and 5′-CTATTGTCTCTAGAAAGTGAG-3′ for LIV-1; 5′-TGCCCAGAAAATGAAAAAGG-3′ and 5′-GTGTATGTGGCAATGCGTTC-3′ for E-cad; ; 5′-CCATCACTCGGCTTAATGGT-3′ and 5′-GATGATGATGCAGAGCAGGA-3′ for N-cad; 5′-CGAAAGGCCTTCAACTGCAAAT-3′ and 5′-ACTGGTACTTCTTGACATCTG-3′ for Snail; 5′-TGCCCGGCGGAATCTCCTGA-3′ and 5′-GATGCAGGAGGGAGCCCGGA-3′ for HB-EGF; 5′- GCTGTCTGCGTGGTGGTGCT -3′ and 5′- TGGTGTGGTGGGTCCAGGGC -3′ for EGF; and 5′-TTAGCACCCCTGGCCAAGG-3′ and 5′-CTTACTCCTTGGAGGCCATG-3′ for GAPDH. The reactions were initiated with a 4-minute incubation at 94°C, followed by 30 cycles at 94°C for 30 seconds, 55°C for 30 seconds, and 72°C for 1 minute. The reaction was completed with a 7-minute extension at 70°C for 7 minutes. PCR products were visualized after electrophoresis through a 1.2% agarose gel and stained by ethidium bromide (0.5 µg/ml).

### Western blotting

Cells at 80% confluence were lysed in a whole-cell lysis buffer as previously reported [22]. The lysates were incubated on ice for 30 minutes and centrifuged at 10,000 rpm at 4°C for 10 minutes. From each sample, 35 µg protein in the supernatant was resolved by SDS-PAGE and blotted onto a nitrocellulose membrane (BioRad, Hercules, CA), which was blocked in 5% skim milk in PBST (137 mM NaCl, 12 mM phosphate, 2.7 mM KCl, and 0.1% Tween 20) at room temperature for 20 minutes and incubated with primary antibody at 4°C overnight. The membranes were then washed three times in PBST and incubated with horseradish peroxidase-conjugated secondary antibody for 1 hour at room temperature. After five washings in PBST, specific signals were detected by incubating the membrane with ECL reagent (Amersham-Pharmacia Biotech, Piscataway, NJ).

### Measuring intracellular zinc concentration

Two methods were used to determine intracellular zinc concentration. To prepare samples for assaying total intracellular zinc by the inductively coupled plasma mass spectrometry (ICP-MS), cultured cells at 80% confluence were trypsinized and washed in PBS, and then incubated in 300 µl of 70% nitric acid at 37°C for 2 hours. The cells were then placed in 2% nitric acid and subjected to ICP-MS with a Varian instrument. A standard curve was generated with serial dilutions of zinc instrument standards. To prepare samples for assaying intracellular labile zinc by fluorometric method, cells were seeded at 3×10^5^ cells per well in 6-well plates the day before measurement. The cells were loaded with 2 µM of FluoZIN-3 AM (Invitrogen) for 1 hour in Opti-MEM containing 0.02% Pluronic F127 (Invitrogen). After washing in PBS, the loaded cells were incubated in indicator-free Opti-MEM for 30 minutes. Fluorescence of the FluoZin-3 was measured using a PE Victor^3^ V plate reader.

### Scratch wound healing assay

ARCaP_M_ cells transfected transiently with LIV-1 siRNA or universal siRNA control were used to determine migratory behavior. The cells were gently and slowly scratched with a 10 µl pipette tip across the center of the well 48 hours after transfection. After scratching, the well was gently washed twice with medium to remove the detached cells. The well was replenished with fresh medium. Photos were taken 48 hrs after scratching. Multiple views of each well were documented, and three independent experiments were performed.

### Trans-well migration and invasion assays

To perform a trans-well migration assay, 2.5×10^4^ cells in the top chamber of 24-well transwell plates of 8 µm pore size (BD Biosciences) were incubated for 16 hours in complete medium that was added to the bottom chamber. Cells were then fixed with formalin and stained with 0.5% crystal violet. The non-migrated cells inside the chamber were removed by swabbing. Crystal violet for the migrating cells was solubilized into Sorenson's buffer (0.1 M sodium citrate and 50% ethanol, pH 4.2) and measured for absorbance at OD_590_. The invasion assay was performed using BD BioCoat Matrigel invasion chambers (BD Biosciences; 8-µm pore size). The same procedures described above were used, except the filters were pre-coated with 100 µl Matrigel at a 1∶4 dilution in RPMI-1640.

### Assessment of tumorigenic and metastatic potentials

The functional roles of LIV-1 in prostate tumor formation and metastasis were assessed as reported previously [23]. To assess local tumor growth, 4-week-old athymic male mice (Ncr-nu/nu, National Cancer Institute, Frederick, MD) were inoculated subcutaneously with ARCaP_E_ cells (1×10^6^ in 50 µl PBS) stably transduced with LIV-1. Tumor dimension was measured with a caliper at days 23, 32, 43, and 50 after injection, and tumor volume was calculated as length × width × height × 0.5236 [24]. To assess cancer metastases, athymic male mice were inoculated intracardiacally with ARCaP_E_ cells (2×10^6^ in 100 µl PBS) stably transduced with LIV-1 to the left ventricles. Animals were observed for 4 months for development of metastatic lesions. Bone metastases were recorded by X-ray radiography and soft tissue metastases were confirmed by histopathology.

### Immunohistochemistry (IHC) of tissue microarray (TMA)

The normal and diseased prostate tissues analyzed were from: 1) One custom-made TMA with normal prostate tissues from 4 healthy men; matched cancer, benign, and PIN tissues from 12 prostate cancer cases; matched benign and cancer tissues from 11 cases; matched PIN and cancer from 1 case; benign prostatic hyperplasia (BPH) from 2 cases; and prostate cancer bony metastasis from 3 prostate cancer cases. 2) One custom-made TMA consisted of 47 bone metastasis tissues from 11 prostate cancer patients. 3) Four TMAs each containing 66 cases of prostate cancer and benign prostate disorders (US Biomax. Rockville, MD).

The IHC protocol for evaluating gene expression has been reported [22]. Briefly, specimens were deparaffinized, rehydrated and subjected to antigen retrieval. After endogenous peroxidase blocking, the specimens were incubated with primary antibody at 4°C overnight, followed by a 30-minute incubation with DakoCytomation EnVision+ HRP reagent. Signals were detected by adding diaminobenzidine as chromogen and counterstaining by hematoxylin. Pre-immunization rabbit serum served as negative control. IHC staining was scored by two investigators independently based on four staining intensities from 0 to +++ as previously reported [22].

### Gelatin zymography

All gelatin zymography reagents were purchased from Invitrogen. Cells were cultured in serum-free RPMI1640 medium for 24 hours and conditioned medium was collected. Protein in the medium was concentrated with an AmiconUltracel 30 KDa filter (Millipore, Billerica, MA). Equal amounts of protein (10 µg/sample) were mixed with 2× Novex Tris-Glycine SDS sample buffer, and fractionated on a 10% gelatin gel under non-reducing conditions. The gel was then incubated at 37°C in renaturing buffer for 30 minutes and in developing buffer for 30 minutes. Finally, the gel was stained in SimplyBlue Safestain, and bands representing the gelatinase activity of MMP2 and MMP9 were quantified.

### Enzyme-linked immunosorbent assay (*ELISA*) for HB-EGF

Cells were cultured in serum-free RPMI1640 medium for 24 hours. Conditioned medium was collected and analyzed for HB-EGF concentration with the Human HB-EGF Duoset ELISA kit (R&D Systems), following the manufacturer's recommended protocol. Each sample from three independent experiments was assayed in triplicate.

### Statistical analysis

To analyze the potential association of LIV-1 protein expression and prostate cancer progression from normal/benign, prostatic intraepithelial neoplasia (PIN), localized primary cancer, to bone metastasis, the LIV-1 expression level was divided into two categories: staining intensity of high (3) vs. medium to null (2 to 0, respectively). The Kruskal Wallis non-parametric test was used to determine the equality of population medians among prostate cancer progressions of normal/benign, PIN, primary cancer, and bone metastasis. This test is equivalent to the parametric ANOVA test used when there are more than two groups being compared. The Mann-Whitney non-parametric test was applied to determine the equality of population medians between two cancer progressions, 1) bone metastasis vs. localized cancer; and 2) bone metastasis vs. benign, PIN, and primary localized cancer. This test is equivalent to the parametric t-test used when there are only two groups being compared. Logistic regression was used to model the relationship between binary Gleason scores which were divided into binary variables of well-differentiated (GI≤6) vs. moderate to poorly differentiated (GI≥7) prostate cancer. SAS and Minitab were used in this analysis.

## Results

The human prostate cancer ARCaP cells established in our laboratory [21] can be readily promoted to undergo EMT in response to soluble factors and matrix proteins present in the tumor microenvironment [20], [25], [26], [27]. To elucidate the molecular mechanism regulating EMT, epithelial ARCaP_E_ was analyzed for differential gene expression in response to soluble factors, in comparison to its ARCaP_M_ counterpart which displayed a mesenchymal phenotype. LIV-1 was one of the differentially expressed genes identified [27]. In the current study, we investigated the role of LIV-1 in regulating EMT in ARCaP cells to assess the possible mechanism of LIV-1 action in the promotion of prostate cancer bone and soft tissue metastases.

### LIV-1 was involved in promoting EMT in the ARCaP cell model

**Figure 1:**
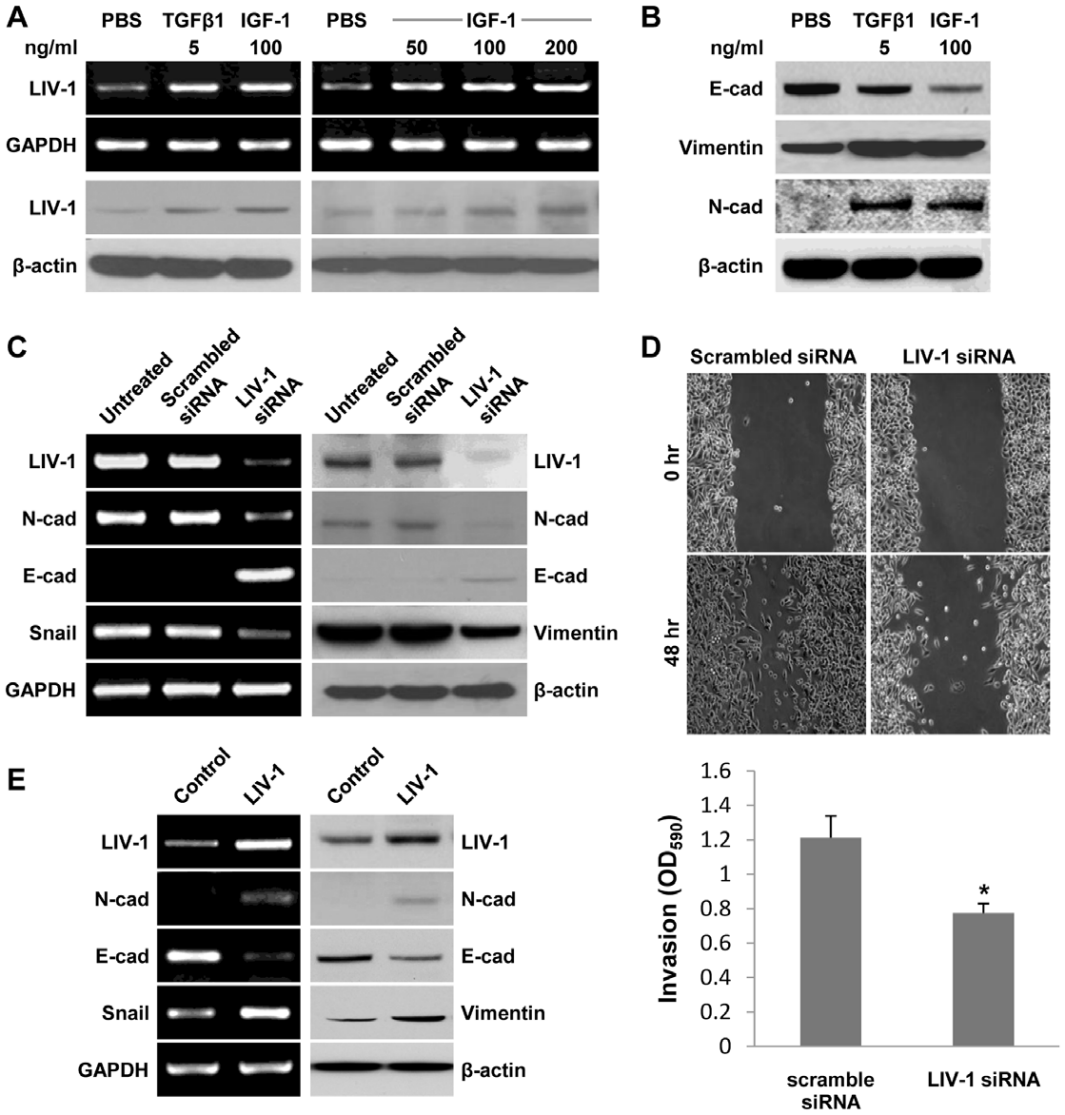
LIV-1 is a mediator in ARCaP_E_ cell EMT. The role of LIV-1 was assessed by its changed expression during EMT. **A,** ARCaP_E_ cells were treated for 48 hours with growth factors to induce EMT. RT-PCR and Western blotting were used to show increased LIV-1 expression (left panel), and the dose responsiveness of the expression (right panel). **B,** Western blotting was used to confirm EMT-like expressional changes in the treated ARCaP_E_ cells. **C,** mesenchymal cell-like ARCaP_M_ cells were subjected to siRNA knockdown for LIV-1 expression for 48 hours. RT-PCR and Western blotting were used to detect expressional changes reflecting reversal of EMT in the treated cells. **D,** Scratch wound healing and transwell invasion assays were used to determine migratory and invasive behavior in siRNA treated ARCaP_M_ cells. * indicates statistical significance compared to the con1 control clone (P<0.05). **E,** ARCaP_E_ cells were transfected with LIV-1 expression construct. RT-PCR and western blotting were performed 48 hours after transfection to detect expressional changes reflecting EMT-like events. GAPDH served as an internal control for RT-PCR reactions, and β-actin was used as a loading control in Western blotting.

We previously reported that ARCaP_E_ cells underwent EMT when treated with soluble factors including IGF-1, EGF, TGF-β1 and β-2 microglobulin (β-2M) [20], [25], [26], [27]. In the present study, when ARCaP_E_ cells were treated with either TGF-β1 or IGF-1, an induction of LIV-1 expression was detected by both RT-PCR and Western blotting analyses (Figure 1A). When different concentrations of IGF-1 were added to the induction medium, the responsiveness of LIV-1 expression was found to be dose-dependent (Figure 1A). IGF-1-induced LIV-1 expression in ARCaP_E_ cells occurred concomitantly with a switch of cell morphology and gene expression toward mesenchymal phenotype, *i.e.*, the loss of tightly adhesive polarized epithelial morphology to become loosely dispersed fibroblastic cells with increased expression of N-cad and vimentin but decreased expression of E-cad, a hallmark retained by polarized epithelial cells (Figure 1B). Activated LIV-1 expression seemed to occur concurrently with the transition of ARCaP_E_ to ARCaP_M_, an ARCaP mesenchymal variant [21].

To define the role of LIV-1 in mediating EMT, we transiently reduced the LIV-1 level in the mesenchymal-like ARCaP_M_ cells by siRNA knockdown. ARCaP_M_ cells treated with specific LIV-1 siRNA showed markedly reduced LIV-1 transcripts (Figure 1C). Importantly, the treated cells showed decreased expression of mesenchymal markers N-cad and Snail, but increased expression of the E-cad gene in both RT-PCR and Western blotting analyses (Figure 1C). In addition, ARCaP_M_ cells treated with specific LIV-1 siRNA exhibited much reduced migratory and invasive ability in scratch wound-healing and transwell invasion assays (Figure 1D). These results suggested that LIV-1 expression is associated with EMT and decreased LIV-1 expression leading to mesenchymal to epithelial transition (MET), a reversal of EMT. The presence of LIV-1 appeared to be required for the maintenance of a mesenchymal phenotype.

We next examined whether elevated LIV-1 in the epithelial-like ARCaP_E_ cells would be sufficient to initiate EMT, as assessed by molecular analyses. Following transient transfection with a LIV-1 expression construct, ARCaP_E_ cells were examined by both RT-PCR and western blotting assays for the expression of EMT-associated markers. The transfected ARCaP_E_ cells displayed markedly increased LIV-1 expression (Figure 1E), accompanied by increased N-cad and Snail but a decreased E-cad expression. These expressional changes were in agreement with those seen in growth factor-elicited EMT (Figure 1B). Results from LIV-1 siRNA knockdown and LIV-1 overexpression studies in ARCaP_M_ and ARCaP_E_ cells suggested that LIV-1 serves a key regulator of EMT in human prostate cancer cells.

### Production and characterization of polyclonal antibodies to human LIV-1

**Figure 2:**
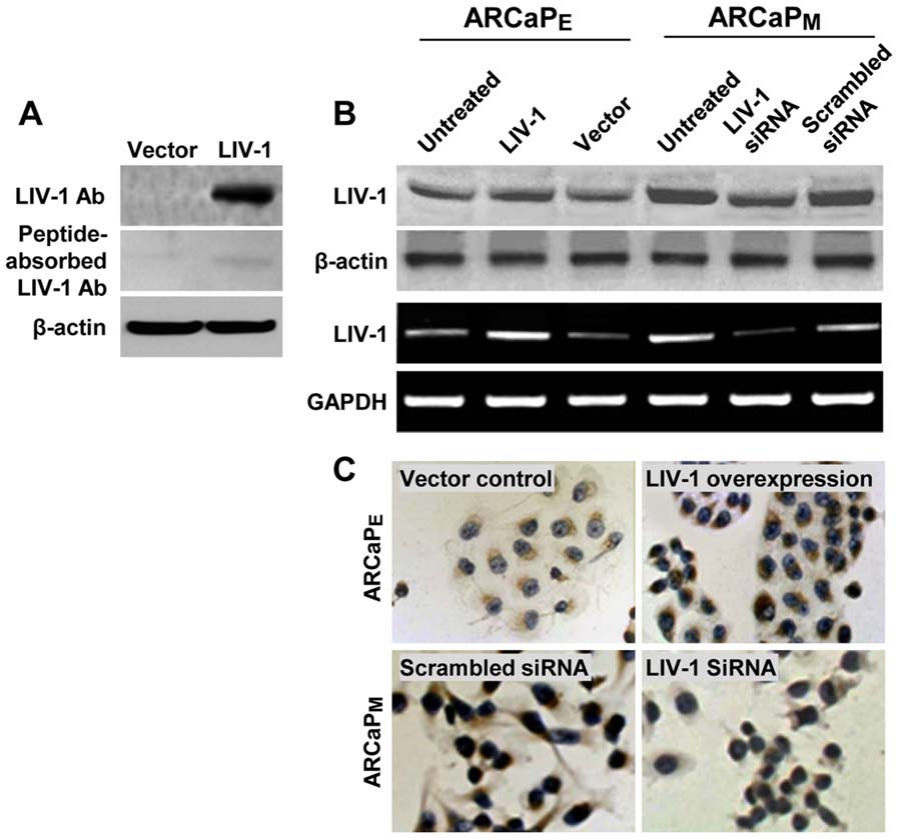
Validation of the antibodies to LIV-1. The produced antibodies to LIV-1 were subjected to validation for specificity. **A,** HEK293 cells transiently transfected with the LIV-1 expression construct were subjected to Western blotting analysis with the antibodies to LIV-1 (upper panel). Antibody specificity was determined by pre-absorbing the antibody with the immunizing peptide (middle panel). **B,** ARCaP_E_ cells were transiently transfected with the LIV-1 expression construct to overexpress LIV-1 and ARCaP_M_ cells with the specific siRNA to suppress LIV-1 expression. In the upper 2 panels, Western blotting was performed 48 hours later with the antibodies to LIV-1. In the lower 2 panels, these cells were examined by RT-PCR to confirm the LIV-1 expression. β-actin was used as control in Western blotting and GAPDH was used as control for RT-PCR analysis. **C,** IHC was conducted to further confirm LIV-1 Ab specificity in ARCaP_E_ cells transiently transfected with the LIV-1 expression construct and ARCaP_M_ cells transiently transfected with the specific siRNA (200 ×).

To evaluate if LIV-1 expression is associated with clinical progression of human prostate cancer, we raised polyclonal antibodies by immunizing rabbits with a KLH-conjugated LIV-1 peptide. Specificity of LIV-1 antibodies was confirmed by Western blotting of the whole-cell extracts from cells overexpressing exogenous LIV-1. From the HEK293 cells transiently transfected with LIV-1, we observed a single immune-reactive LIV-1 protein, at 110 kDa (Figure 2A). Since the calculated molecular weight of LIV-1 protein is 90 kDa [5], the differential 20 kDa between the detected and the predicted sizes was likely attributed to N-linked glycosylation of the LIV-1 protein, as previously reported [5]. Importantly, the signal detected by the LIV-1 antibodies was abolished when the antibodies were pre-adsorbed with the LIV-1 peptide used in immunization. In addition, increased signal intensity was detected in ARCaP_E_ cells transiently transfected with the LIV-1 expression construct, while a reduction of the signal was seen in ARCaP_M_ cells treated with a transient LIV-1 knockdown vector in both Western blotting and IHC assays (Figure 2B and 2C). These results indicated that the LIV-1 antibodies produced could detect specifically LIV-1 protein, which was modified in the cell lines tested.

### Stable LIV-1 overexpression induced EMT in ARCaP_E_ cells

Following transient knockdown of LIV-1 in ARCaP_M_ cells, an expected reversal of the mesenchymal fibroblastic cell shape to epithelial morphology was observed. These morphologic switches were readily detectable by gene expression changes (Figure 1C). In contrast, transiently overexpressing LIV-1 in ARCaP_E_ cells did not induce mesenchymal morphologic transition, despite concerted expressional changes indicative of EMT (Figure 1E). We suspected that the lack of morphologic changes may be attributable to the nature of the transient transfection. Accordingly, stable ARCaP_E_ clones were established to evaluate whether LIV-1 is a critical regulator associated with morphologic as well as expressional and behavioral transition from an epithelial to a mesenchymal phenotype.

**Figure 3:**
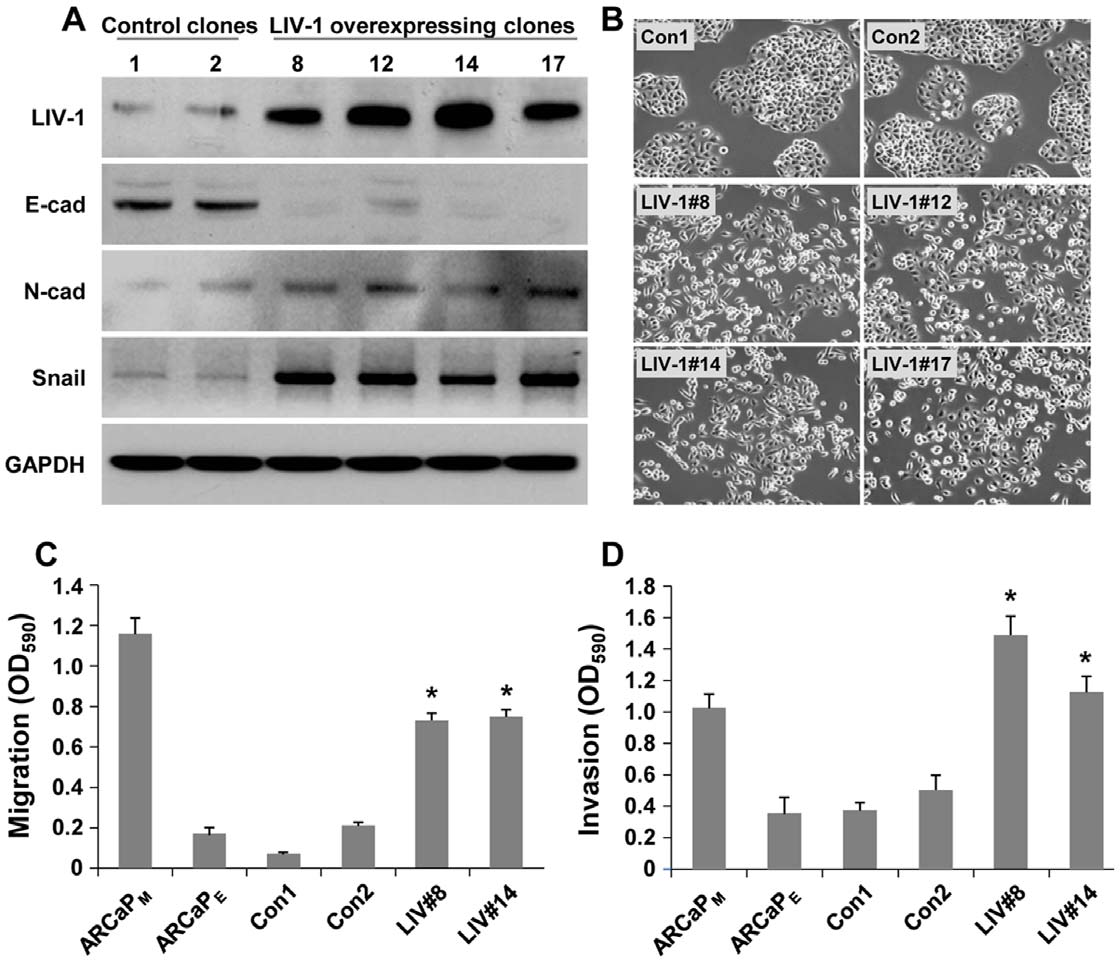
LIV-1 overexpression induced EMT. ARCaP_E_ clones overexpressing LIV-1 displayed EMT-like changes in gene expression, cellular morphology and behavior. **A,** all four LIV-1 overexpressing ARCaP_E_ clones showed EMT-like expressional changes as detected by Western blotting, while the two vector control clones (1 and 2) retained an epithelial cell-like expression profile. **B,** cellular morphology of the LIV-1 overexpressing cells showed marked changes from the control clones (200 ×). **C,** LIV-1 overexpressing cells (LIV#8 and LIV#14 were compared with vector control clones 1 and 2 and parental ARCaP_E_ and ARCaP_M_ cells for altered migratory capability in transwell assays. Each result is the mean ± standard deviation of a triplicate assay. **D,** the LIV-1 overexpressing #8 and #14 clones were compared with vector control clones 1 and 2 and parental ARCaP_E_ and ARCaP_M_ cells for altered invasiveness. * indicates statistical significance compared to the con1 control clone (*P*<0.05).

We isolated 4 ARCaP_E_ clones (LIV#8, 12, 14 and 17) stably expressing high levels of LIV-1 protein, as detected by Western blotting (Figure 3A). Two control clones (con1 and con2) were also isolated from transfection with the control vector. The ones overexpressing LIV-1 showed typical EMT-like expressional changes, with decreased E-cad expression but increased N-cad and Snail expressions (Figure 3A). Significantly, all the clones showed markedly changed cellular morphology: instead of the small cell size with cobblestone-like shape with tightly arranged intercellular contact typical of the epithelial cell-like ARCaP_E_, all four clones adapted remarkably altered morphology displaying a loss of intercellular contact and typical spindle-shaped mesenchymal cell morphology (Figure 3B). The morphologic transition was permanent and irreversible, persisting after more than 30 passages in continuous culture, while the two vector-transfected clones remained epithelial cell-like. It seems that stable LIV-1 overexpression could bring forth both morphologic and biochemical EMT transition. LIV-1 is thus a potent promoter of EMT in ARCaP_E_ cells.

The effects of LIV-1 on behavioral changes were assessed for its promotion of cell migration and invasion in Boyden chamber assays. While the control neo transfected ARCaP_E_ clones showed similar migration and invasion capabilities closely mimicking those of the parental ARCaP_E_ cells, repeated assays revealed that LIV-1 overexpression conferred significantly increased migratory capability (Figure 3C) and invasive potential to penetrate extracellular matrices (Figure 3D). Taken together, these data support the notion that increased LIV-1 levels promote the motility and invasive behaviors of prostate cancer cells.

### LIV-1 overexpression promoted prostate tumor formation and distant metastases *in vivo*

We examined the role of LIV-1 stably expressed in ARCAP_E_ cells in modulating subsequent tumorigenic and metastatic behaviors in mice. We compared local and distant metastatic growth of ARCaP_E_ tumors by subcutaneous and intracardiac tumor cell inoculation protocols as described previously [21], [23].

**Figure 4:**
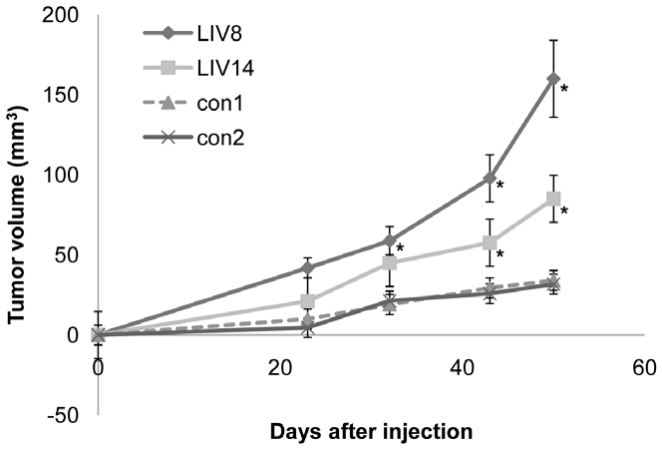
LIV-1 overexpression promoted subcutaneous tumor growth. Upon subcutaneous inoculation, the two ARCaP_E_ clones overexpressing LIV-1 (LIV8 and LIV14) were compared with vector control clones (con1 and con2) for tumor formation in athymic mice. Growth of the tumors at day 23, 32, 43, and 50 is shown. Each result represents the mean ± standard deviation of six tumors. An asterisk indicates statistical significance compared to the con1 control clone (*P*<0.05).

Following subcutaneous implantation, LIV-1 overexpressing clones induced a similar incidence of tumor formation to the vector-transfected controls, each group having 6 tumors from a total of 8 inoculations. Nonetheless, LIV-1-overexpressing clones formed significantly larger tumors than the control clones when the tumors were measured at 43 and 50 days after inoculation (Figure 4). Due to the large tumor burden in the LIV-1 transfected experimental group, these studies were terminated at day 50. At this time, the average tumor size of LIV-1-overexpressing clones was 3 - 5 times larger than that of the control clones, with no evidence of distant metastases.

**Figure 5:**
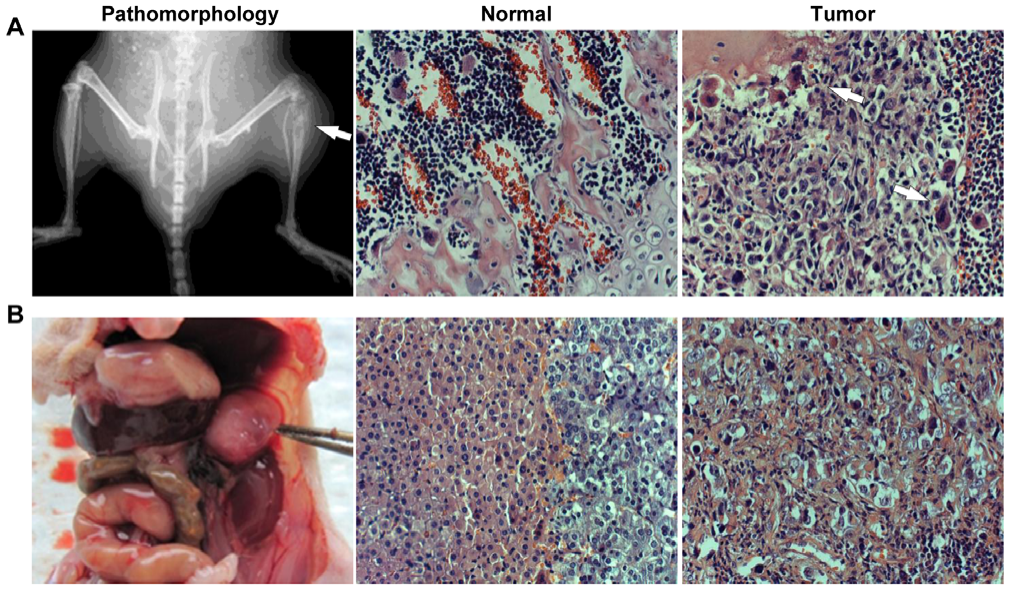
LIV-1 overexpression promoted cancer bone and soft tissue metastasis. Upon intracardiac inoculation, ARCaP_E_ clones overexpressing LIV-1 caused metastatic tumor formation in athymic mice (Table 1). Representative results depict the metastatic tumors. **A,** a tibial tumor (arrow) was identified by X-ray (Pathomorphology). The tumor was subjected to H&E staining for histopathologic confirmation. Compared to the tibia of the opposite leg (Normal), the tibial tumor (Tumor) showed histopathology typical of a metastatic bone lesion. **B,** an adrenal gland metastasis (held by forceps) destroyed the gland (Pathomorphology). Compared to the unaffected gland (Normal), tumor cells were found in every part of the affected gland (Tumor) (250 ×).

We then used intracardial inoculation to evaluate the metastatic fate of LIV-1 overexpressing ARCaP_E_ cells. Four months after intracardiac administration, the mice inoculated with LIV-1 overexpressing ARCaP_E_ clones presented with significantly elevated incidence of tumors at multiple organ sites, including the bone and soft tissues of lymph nodes, adrenal glands and lung, compared to vector-transfected controls (Table 1). Among the seven animals inoculated with LIV-1-overexpressing ARCaP_E_ clone 8, two were found to have multiple bone metastases, both in tibial, mandibular, and spinal bones (Figure 5A), while another four mice were found to harbor soft tissue tumors of the adrenal glands and the lung (Figure 5B). All the tumors were confirmed by histopathologic analysis. In a parallel study, five of the nine animals inoculated with LIV-1-overexpressing ARCaP_E_ clone 14 were found to bear bone metastasis. In sharp contrast, intracardiac inoculation of vector-transfected controls did not produce any detectable metastases in bone or soft tissues (Table 1). This series of assays demonstrated that by increasing the migratory and invasive behaviors of prostate cancer cells, LIV-1 promoted metastatic growth of prostate cancer cells to the bone and the soft tissues.

**Table 1:** LIV-1 promotes prostate cancer cell metastasis.*

Clones	Incidence of metastasis	Sites of metastasis
con1	0/6	N/A
con2	0/6	N/A
LIV#8	6/7 (86%)	2, adrenal gland
		2, lung
		1, leg bone
		1, jaw and spine
LIV#14	5/9 (56%)	2, leg bone
		1, iliac bone
		1, femur
		1, zygomatic and spine

*LIV-1-overexpression clones (LIV#8 and LIV#14) and vector-transfected clones (con1 and con2) were intracardially inoculated to athymic mice. Incidence and sites of metastasis were followed up to 4 months by necropsy and histopathologic confirmation.

### Enhanced LIV-1 expression in clinical prostate cancer specimens

**Figure 6:**
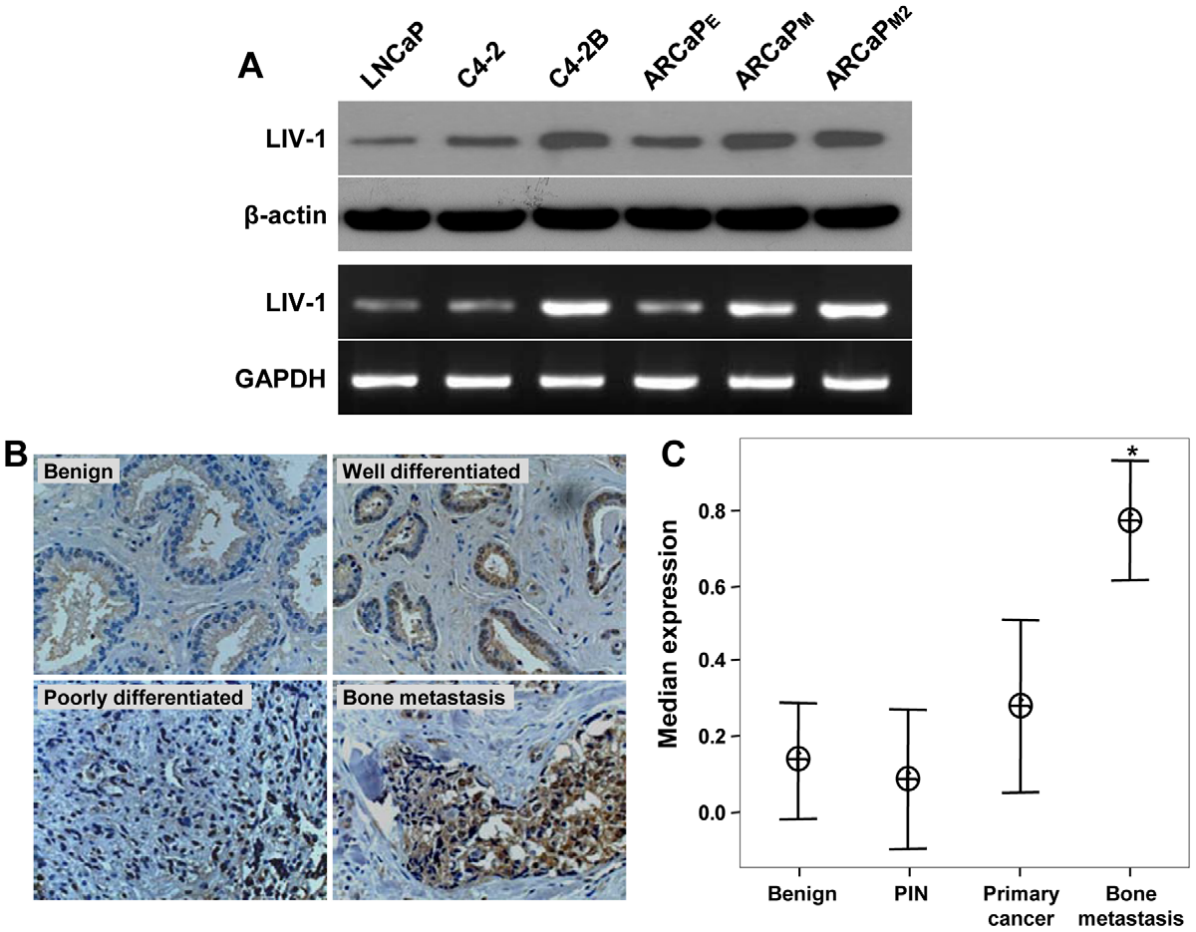
LIV-1 expression is associated with human prostate cancer progression. **A,** RT-PCR and Western blotting were used to determine LIV-1 expression in different prostate cancer cell lines. β-actin was used as control in Western blotting and GAPDH was used as control for RT-PCR analysis. **B,** representative IHC images showed increased LIV-1 expression in human prostate specimens from benign to bone metastasis (125 ×). **C,** Interval plot of LIV-1 expression is shown versus prostate cancer progression from normal/benign, PIN, primary cancer to bone metastasis. The data are shown with 95% confidence interval (n  =  number of cases analyzed). The median expression for LIV-1 in bone was significantly greater than in normal/benign, PIN, and primary cancer (*P*<0.001) and in primary cancer only (P = 0.002) as analyzed by Mann-Whitney test.

In order to examine the correlation between LIV-1 expression and cancer malignancy, we first assessed LIV-1 expression in different prostate cancer cell lines. We chose the isogenic LNCaP series (LNCaP, C4-2 and C4-2B) and ARCaP series (ARCaP_E_, ARCaP_M_ and ARCaP_M2_) cell lines. Both series range from low potential to high potential for metastasis. C4-2, C4-2B, ARCaP_M_ and ARCaP_M2_ specifically metastasize to bone. We found that LIV-1 expression correlated with the metastatic potential of these isogenic cell models. LIV-1 expression is higher in the bone metastatic cell lines than non- or low-metastatic cells such as LNCaP and ARCaP_E_ (Figure 6A).

Based on findings in ARCaP cells where LIV-1 was shown to induce EMT and prostate cancer local growth and distant metastases and LIV-1 expression in different cancer cell lines, we sought to demonstrate the clinical relevance of these findings by performing a series of IHC assays. A LIV-1 polyclonal antibody established by our laboratory was used to detect LIV-1 status in clinical specimens on two custom-made TMAs of normal, benign/PIN, localized and bone metastatic prostate cancers and four commercial TMAs of benign and prostate cancer specimens. These TMAs represented a total of 344 prostate specimens including bone metastasis specimens from 14 patients. Results from a Kruskal Wallis non-parametric test showed a remarkable positive correlation of LIV-1 expression with disease progression from normal, benign, PIN, and primary to bone metastasis (*P*<0.001). Figure 6B shows representative IHC images of increased LIV-1 expression in human prostate specimens from benign to bone metastasis. Significant differences were also found when LIV-1 expression was subjected to Mann-Whitney non-parametric test between either bone metastasis versus localized cancer (p = 0.002) or bone metastasis versus normal/benign, PIN, primary cancer and metastasis (p = 0.001) (Figure 6C). There was, however, no positive correlation between LIV-1 expression in well-differentiated (GI≤6) and moderate to poorly-differentiated (GI≥7) prostate cancers by Logistic regression test.

### LIV-1 overexpression activated EGFR and downstream ERK signaling

**Figure 7:**
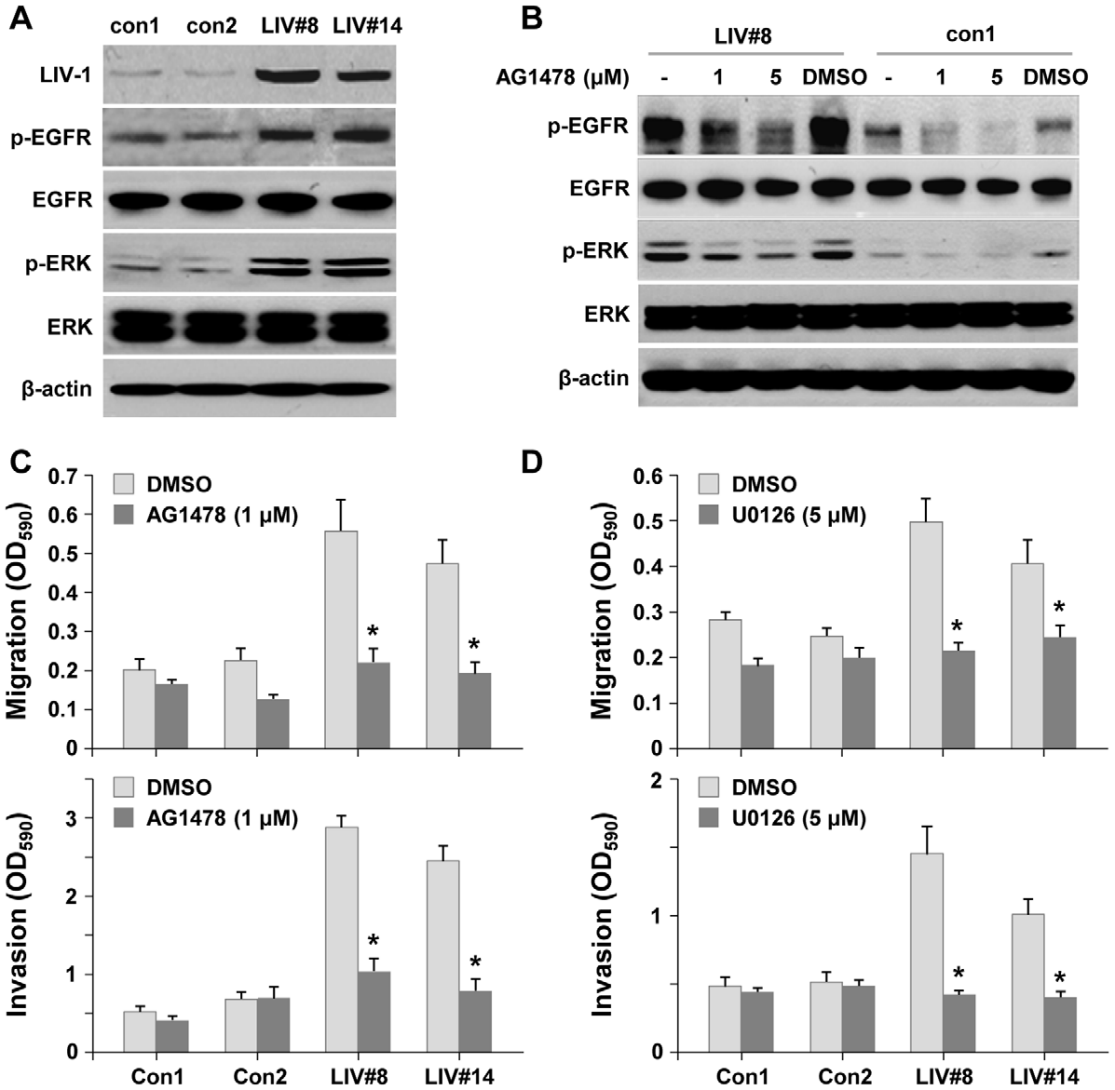
LIV-1 overexpressing cells displayed constitutively activated EGFR signaling. A, LIV-1 overexpressing cells (LIV#8 and LIV#14) showed increased phosphorylated EGFR (p-EGFR) and ERK (p-ERK). **B,** EGFR inhibitor (AG1478) treatment reduced phosphorylated EGFR and ERK in LIV#8 clone. **C,** inhibition of EGFR suppressed migratory ability and invasive ability of LIV-1 overexpressing clones in transwell assays 24 hours after treatment. **D,** inhibition of ERK signaling resulted in similar suppression of cellular motility as EGFR inhibition. * indicates statistical significance compared to the control of the same group (*P*<0.05).

We next explored the mechanism by which LIV-1 promoted prostate cancer EMT, progression and metastasis. We examined the phosphorylation status of AKT, p38, JNK, Smad, NF-κB, β-catenin and ERK because these regulatory proteins were shown to be altered by a soluble growth factor, β2-M, which also promoted EMT and LIV-1 expression [25], [26], [27]. In this study, we observed that ERK signaling was significantly activated (Figure 7A). Since ERK was frequently activated by growth factor receptor signaling, we examined specifically the phosphorylation status of IGF-1R, TGF-β receptor and EGFR proteins. This series of analyses revealed specifically increased EGFR phosphorylation in the LIV-1-overexpressing cells (Figure 7A). To elucidate the relationship between EGFR and ERK activation, we used the specific inhibitor AG1478 to block EGFR activation. Inhibition of EGFR phosphorylation led to a simultaneous reduction in ERK phosphorylation (Figure 7B), suggesting that EGFR activation is responsible for downstream ERK phosphorylation.

We examined next whether EGFR-induced ERK activation was responsible for the increased metastatic behaviors of LIV-1 overexpressing ARCaP_E_ cells in transwell migration and invasion assays. Blocking EGFR activation by AG1478 was accompanied by reduced migratory and invasive capabilities (Figure 7C). In support of these results, ERK inhibitor U0126 treatment also reduced both the migratory and invasive capabilities (Figure 7D), similar to AG1478 treatment. These results, taken together, demonstrated that EGFR and downstream ERK activation is the major signaling pathway stimulating the migratory and invasive behavior of LIV-1 overexpressing cells.

### LIV-1 activated EGFR signaling by increasing HB-EGF, MMP2 and MMP9 expression

Since LIV-1 is a member of the ZIP metal transporters that may regulate intracellular zinc ion homeostasis [1], [5], [28], we evaluated whether the increased EGFR constitutive phosphorylation in LIV-1 overexpressing clones was due to changes in intracellular zinc concentration. Compared to the control clones, however, there were no statistically significant differences in both the total zinc determined by ICP-MS analysis and the labile fraction measured by the fluorometric assay method (data not shown).

We then tested the hypothesis that as a result of activation of EGFR in LIV-1 overexpressing cells, a positive feedback consisting of an autocrine/paracrine loop of growth factors may be elicited to account for increased tumorigenicity and metastatic potential. We examined whether the increased EGFR phosphorylation was a result of increased production of cognate ligands such as EGF and HB-EGF proteins [29], [30].

**Figure 8:**
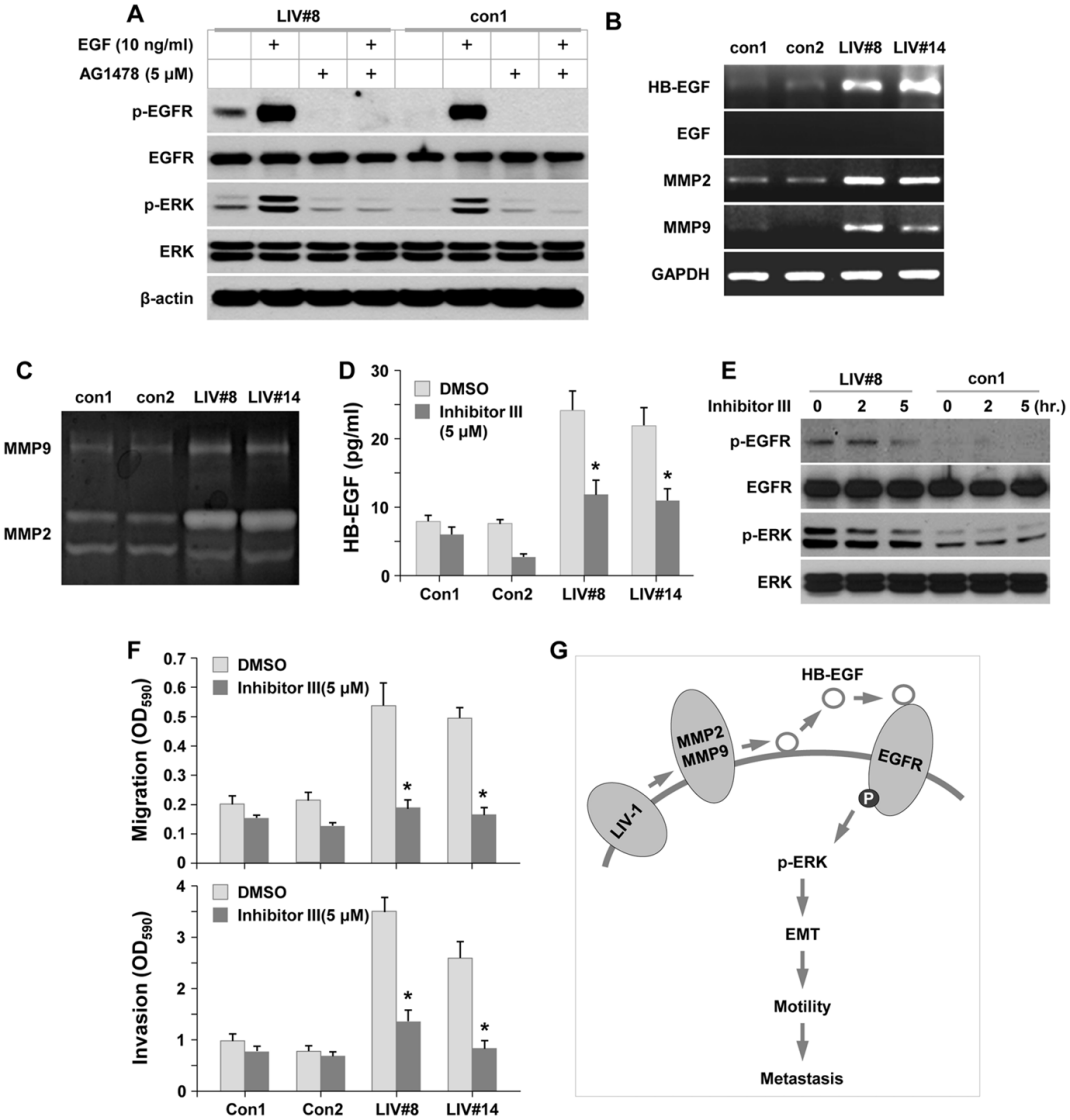
HB-EGF level and MMP2/9 activity were up-regulated in LIV-1 overexpressing cells. **A,** LIV-1 overexpressing cells (LIV#8) and control cells (con1) were treated with EGF and AG1478 for 2 hours. Western blotting showed that the EGF-elicited EGFR and ERK phosphorylation was blocked by the AG1478 inhibitor. **B,** RT-PCR showed increased HB-EGF, MMP2 and MMP9 expression in LIV-1 overexpressing cells. **C,** LIV-1 overexpressing cells (LIV#8 and LIV#14) were cultured in serum-free medium for 24 hours and the culture media were used to determine the MMP2 and MMP9 enzymatic activity by zymogram assay. **D,** the effect of MMP2/9 enzymatic activity on HB-EGF shedding was evaluated by ELISA. LIV-1 overexpressing clones (LIV#8 and LIV#14) secreted more HB-EGF than control clones (con1 and con2), and the secretion was reduced by MMP2/9 inhibition. **∗** indicates statistical significance compared to the control of the group (*P*<0.05). **E,** Western blotting showed that inhibition of MMP2/9 activity suppressed EGFR and the downstream ERK phosphorylation. **F,** LIV-1 overexpressing cells were treated with MMP 2/9 inhibitor III for 24 hours in Transwell motility assays. Both migration and invasion of the treated cells were decreased. * indicates statistical significance compared to the control group (*P*<0.05). **G,** diagram depicts the proposed role of LIV-1 in prostate cancer cell EMT and metastasis.

Western blotting revealed that LIV-1 overexpressing ARCaP_E_ clones and the control clones had similar levels of EGFR protein, and upon ligand treatment both clones could be drastically phosphorylated, which was abolished by specific inhibitor AG1478 (Figure 8A). Nonetheless, LIV-1 overexpressing LIV-1 cells showed a constitutive EGFR phosphorylation in the absence of exogenous ligand, and this phosphorylation could be abolished by AG1478 treatment (Figure 8A). To investigate the cause of the constitutive EGFR phosphorylation, we found while both of these cell types produced low or undetectable levels of EGF by RT-PCR, significantly increased levels of HB-EGF were expressed by LIV-1 overexpressing clones compared to the control clones (Figure 8B). These results indicated that HB-EGF may be a constitutive inducer for EGFR signaling via increased EGFR phosphorylation.

HB-EGF is synthesized as a membrane-anchored form which needs to be released from the plasma membrane by matrix metalloproteases (MMPs) in order to bind EGFR [31], [32], [33]. Interestingly, there was significantly higher MMP2 and MMP9 expression in the LIV-1-overexpressing clones (Figure 8B), while gelatin zymography demonstrated that MMP2 and MMP9 enzymatic activities were also enhanced (Figure 8C). Importantly, treatment with MMP2/9 inhibitor III led to a significant reduction of soluble HB-EGF in the culture media as determined by ELISA (Figure 8D), suggesting that these proteolytic enzymes were involved in HB-EGF shedding. In other experiments, MMP2/9 inhibitor III treatment caused a time-dependent loss of phosphorylation in both EGFR and the downstream ERK proteins (Figure 8E), confirming that the proteolytic enzymes acted upstream of EGFR-elicited MAPK signaling. Consequently, MMP2/9 inhibition significantly reduced the migratory and invasive ability of LIV-1-overexpressing cells in transwell assays (Figure 8F). It seemed likely that the function of LIV-1 was to stimulate the expression of MMP2, MMP9 and HB-EGF proteins, which in turn activated EGFR and downstream ERK signaling, leading to EMT that facilitated local tumor growth and its distant metastases to bone and soft tissues (Figure 8G).

## Discussion

Using the well-characterized ARCaP human prostate cancer progression model, we found that LIV-1 is involved in the promotion of prostate cancer cell EMT, local growth and distant metastases. This conclusion is based on the following evidence: 1) the induction of EMT by IGF-1 or TGF-β1 in ARCaP_E_ cells was accompanied by elevated LIV-1 expression (Figure 1A and 1B); 2) LIV-1 expression was elevated in the isogenic cells expressing mesenchymal phenotype, *i.e.* higher LIV-1 expression was found in ARCaP_M_ compared to ARCaP_E_ cells (Figures 1C, 1E and 6A); 3) overexpression of LIV-1 in ARCaP_E_ cells promoted irreversible EMT of these cells, leading to increased local growth and distant metastases to bone and soft tissues (Figures 3, 4, 5); 4) certain EMT-inducing growth factors, such as β2-M, could both activate LIV-1 expression and promote bone and soft tissue metastases in prostate, breast, lung and renal cancer cells [26]; and 5) selected repression of LIV-1 in ARCaP_M_ cells was accompanied by a reversal of EMT, causing ARCaP_M_ to adopt a phenotype similar to that of the ARCaP_E_ cells with decreased migration and invasion (Figure 1C and 1D). In addition, the function of LIV-1 in the promotion of aggressive cancer behaviors is also supported by data obtained from the analyses of a large number of clinical prostate cancer specimens (Figure 6B and 6C).

Prostate cancer cells in metastasized tumors are known to display both epithelial and mesenchymal phenotypes including morphology and gene expression profiles, and metastatic prostate tumors are likely comprised of heterogeneous populations of both epithelial and mesenchymal cells. With regard to the mechanism of interconversion between epithelial and mesenchymal phenotypes through EMT and MET, it is interesting to note that increased LIV-1 expression can be achieved by hormonal induction [3], [8] and by growth factor engagement (Figure 1). It is conceivable that with induction of LIV-1, cancer cells can establish metastatic foci through EMT that confers increased migratory and invasive capabilities. Inside the tumor metastasis, LIV-1 expression may subside once the inducer withdraws, leading to tumor colonization with cancer cells resuming epithelial morphology and biomarker expression.

Since LIV-1 overexpression was associated with the development of larger prostate tumors (Figures 4 and 5) and with accelerated proliferation *in vitro* (data not shown), we carried out mechanistic analyses to elucidate the underlying regulatory pathway. LIV-1 overexpressing clones expressed high levels of HB-EGF (Figure 8B and 8D). At the same time, these clones produced significantly increased MMP2 and MMP9 transcripts (Figure 8B) as well as enzymatic activities (Figure 8C). The enzymatic activities are involved in catalyzing HB-EGF shedding (Figure 8D). The soluble form of HB-EGF may interact with EGFR to cause constitutive EGFR phosphorylation (Figure 8E). EGFR phosphorylation leads to ERK-mediated signaling transduction, which favors cell growth and facilitates cellular motility. In addition, ERK-mediated signaling may promote EMT by downregulating E-cad expression, thus releasing β-catenin from cytoplasmic membrane to enter the nucleus, where β-catenin interacts with T cell-factor/lymphoid enhancer factor (LEF) transcription factors to promote the growth and survival of cancer cells [34].

The close correlation between LIV-1 level and cancer progression documented in the clinical specimens is likely a general phenomenon in prostate cancer. Several factors may contribute to abnormally enhanced LIV-1 expression during prostate cancer progression and metastasis. It has been shown that LIV-1 expression is negatively regulated by intracellular zinc concentration [1], [5], which provides an auto-regulatory negative feedback [1]. Prostate tumor cells are frequently observed with a lowered intracellular zinc pool [17], [35], [36], which may induce LIV-1 expression. Additionally, loss of intracellular zinc may prevent cancer cells from apoptotic death, since a lowered zinc level can alter mitochondrial membrane potential to hamper the release of apoptosis-triggering caspases [37]. On the other hand, LIV-1 expression may also be stimulated by growth factors in the tumor microenvironment, since treatment with TGFα, TGF-β1, EGF, IGF-1 and β2-M all enhanced the LIV-1 level [3], [4], [27]. However, no direct evidence shows that LIV-1 overexpression by genetic or epigenetic mechanisms leads to cancer progression. We reported recently that β2-M-mediated signaling could lead to a decreased intracellular iron which drives EMT and cancer lethality to bone and soft tissues [26]. Currently it is not clear if LIV-1 is involved in an autoregulatory loop in the regulation of intracellular zinc and iron. In this study, we did not find differences in intracellular total zinc or labile zinc concentrations between LIV-1 overexpressed and neo-control ARCaP_E_ cells, suggesting that LIV-1 overexpression did not affect the intracellular zinc pool in prostate cancer cells. Additional studies should be carried out to determine the role of LIV-1 in determining zinc transport in other prostate cancer cell lines.

Growth factor regulation by LIV-1 could be mediated by STAT3 [15], which orchestrates the nuclear translocation of several important pleiotropic transcription factors. LIV-1 was shown to increase Snail transcription, translation and translocation to cell nucleus in zebrafish [15]. Among many of the common downstream transcription factors responsive to the pleiotropic signals, Snail functions to drive EMT [38]. By directly repressing E-cad transcription, Snail decreases cellular polarity and cell-cell junction but promotes EMT not only in cancer cells, but also in wound healing and renal fibrosis [7], [18], [19]. The relationship between Snail and LIV-1 expression has been reported in breast, cervical and pancreatic cancer progression and lymph node metastases [8], [12], [13], [14], [39]. EMT plays a pivotal role in cell motility during embryonic development [18], [40], while breast cancer cells undergoing EMT can gain stem cell-like properties with increased ability for self renewal as determined by anchorage-independent growth [41]. The link between EMT and stem cell-like properties could have broader clinical implications for the development of novel therapeutic approaches for cancer. The pathophysiologic roles of LIV-1, however, may be cell context-dependent as revealed in some breast cancer cell lines, LIV-1 expression has been associated with the suppression of E-cad, while in others, LIV-1 knockdown paradoxically increased cancer invasiveness [42].

Our results as described herein emphasize a coordinated regulation of LIV-1 expression during prostate cancer cell EMT which ultimately confers increased migratory, invasive and metastastic potential. LIV-1 expression increased EGFR-ERK signaling, through the shedding of HB-EGF from cell surface, by a concomitant induction of MMP2 and MMP9 proteolytic enzymes, which cleave the membrane-bound HB-EGF. The soluble HB-EGF is responsible for EGFR phosphorylation and downstream ERK signaling. Constitutive EGFR activation is a common oncogenic signal in prostate cancer as well as in other malignancies. Our study established for the first time a close link between LIV-1 expression and EGFR-ERK signaling which drives EMT and prostate cancer migration, invasion and metastases. LIV-1 could be a new biomarker and a new therapeutic target for prostate cancer progression and metastasis.
